# NMDA Receptor Antagonists for Treatment of Depression

**DOI:** 10.3390/ph6040480

**Published:** 2013-04-03

**Authors:** Zeynep Ates-Alagoz, Adeboye Adejare

**Affiliations:** 1 Department of Pharmaceutical Sciences, Philadelphia College of Pharmacy, University of the Sciences in Philadelphia, 600 South 43rd Street, Philadelphia, PA 19104, USA; 2 Department of Pharmaceutical Chemistry, Faculty of Pharmacy, Ankara University, Tandogan 06100, Ankara, Turkey

**Keywords:** NMDA receptor antagonists, depression, antidepressant-like effect, ketamine, MK-801, forced swim test

## Abstract

Depression is a psychiatric disorder that affects millions of people worldwide. Individuals battling this disorder commonly experience high rates of relapse, persistent residual symptoms, functional impairment, and diminished well-being. Medications have important utility in stabilizing moods and daily functions of many individuals. However, only one third of patients had considerable improvement with a standard antidepressant after 2 months and all patients had to deal with numerous side effects. The *N*-methyl-d-aspartate (NMDA) receptor family has received special attention because of its critical role in psychiatric disorders. Direct targeting of the NMDA receptor could result in more rapid antidepressant effects. Antidepressant-like effects of NMDA receptor antagonists have been demonstrated in different animal models. MK-801 (a use-dependent channel blocker), and CGP 37849 (an NMDA receptor antagonist) have shown antidepressant properties in preclinical studies, either alone or combined with traditional antidepressants. A recent development is use of ketamine clinically for refractory depression. The purpose of this review is to examine and analyze current literature on the role of NMDA receptor antagonists for treatment of depression and whether this is a feasible route in drug discovery.

## 1. Introduction

Major depression is a psychiatric disorder that affects millions of people worldwide. The disease has serious implications in terms of quality of life and economic burdens associated with loss of work and health-care costs. Depressed patients have high susceptibility for suicide, in part due to complications arising from stress. Depression is projected to be the second leading cause of death by the year 2020 [1]. Individuals that have depression commonly experience high rates of relapse, persistent residual symptoms, functional impairment, and diminished well-being [2,3,4]. Current medications for depression try to increase the level of biogenic amines such as norepinephrine (NE), dopamine (DA) and serotonin (5HT) by a variety of mechanisms including inhibition of degradation or blocking reuptake of the neurotransmitters [5,6]. Medications have had important utility in stabilizing moods and daily functions of many individuals. However, only one third of patients had considerable improvement with a standard antidepressant after 2 months and all patients had to deal with numerous side effects [7,8].

The NMDA receptor has been implicated in the pathophysiology of a variety of neurological and neuropsychiatric diseases including Alzheimer's disease [9], epilepsy, chronic pain syndrome, schizophrenia, Parkinson's disease, Huntington's disease [10,11], major depression, addiction, and anxiety [12]. Excessive glutamate and subsequent over-stimulation of NMDA receptors leading to excessive Ca^2+^ influx has been implicated in the pathophysiology of many neurodegenerative diseases [13,14]. NMDA receptor family also has received special attention because of its critical role in psychiatric disorders [15,16,17,18,19]. Direct targeting of the NMDA receptor could result in an alternative strategy to treat depression.

Antidepressant-like effects have been demonstrated by several types of NMDA receptor antagonists in different animal models [20,21,22,23,24,25]. These antagonists include competitive and non-competitive, antagonists and partial agonists at strychnine insensitive glycine receptors, and also antagonists acting at polyamine binding sites. MK-801 (a use-dependent channel blocker or uncompetitive antagonist), and CGP 37849 (a competitive antagonist) have shown antidepressant properties in preclinical studies, either alone or combined with traditional antidepressants [26,27,28,29,30,31]. Ketamine is a non-competitive NMDA antagonist and a derivative of PCP which was found to produce rapid, robust and persistent antidepressant effects clinically [32]. Therefore, it appears that NMDA receptor antagonists may be key to developing a new generation of improved treatments for major depression. In this review, we describe recent advances in this area, examined and analyzed current literature on the role of NMDA receptor antagonists for treatment of depression.

## 2. NMDA Receptor and Subunits

Glutamate receptors are classified into three subtypes: α-amino-3-hydroxy-5-methyl-4-isoxazolepropionate (AMPA), *N*-methyl-d-aspartate (NMDA) and metabotropic and heterotrimeric GTP-binding protein-linked glutamate receptors (mGluRs) [33]. *N*-methyl-D-aspartate receptors (NMDARs) have attracted particular attention because of their involvement in depression. NMDARs constitute a subfamily identified by specific molecular composition and unique pharmacological and functional properties [34,35,36]. Of particular importance is the high permeability to calcium ions, which confers on NMDARs a central role in both synaptic plasticity under physiological conditions and neuronal death under excitotoxic pathological conditions. Because they are built by heteromeric assembly from a relatively large pool of homologous subunits, NMDARs exist as diverse subtypes endowed with distinctive functional properties and patterns of expression [37].

NMDARs are heteromeric complexes incorporating different subunits to form three subtypes: NR1, NR2 and NR3 (GluN1, GluN2, and GluN3). There are eight different GluN1 subunits generated by alternative splicing from a single gene, four different GluN2 subunits (A, B, C and D) and two GluN3 subunits (A and B); the GluN2 and GluN3 subunits are encoded by six separate genes [34,35,38]. Expression of functional recombinant NMDARs in mammalian cells requires the co-expression of at least one GluN1 and one GluN2 subtype. The stoichiometry of NMDARs has not yet been established definitively, but the consensus is that NMDARs are tetramers that most often incorporate two GluN1 and two GluN2 subunits of the same or different subtypes [34,35,38]. In cells expressing GluN3, it is thought that this subunit co-assembles with GluN1 and GluN2 to form ternary GluN1/GluN2/GluN3 tetrameric complexes [39].

## 3. NMDA Receptor Antagonists

**Figure 1:**
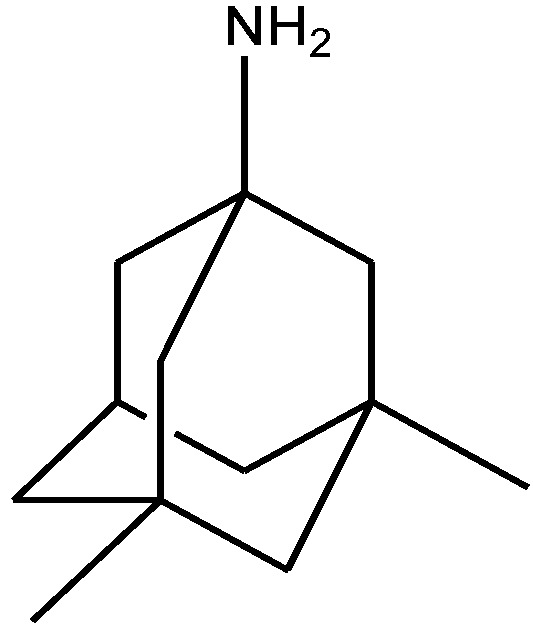
Memantine.

**Figure 2:**
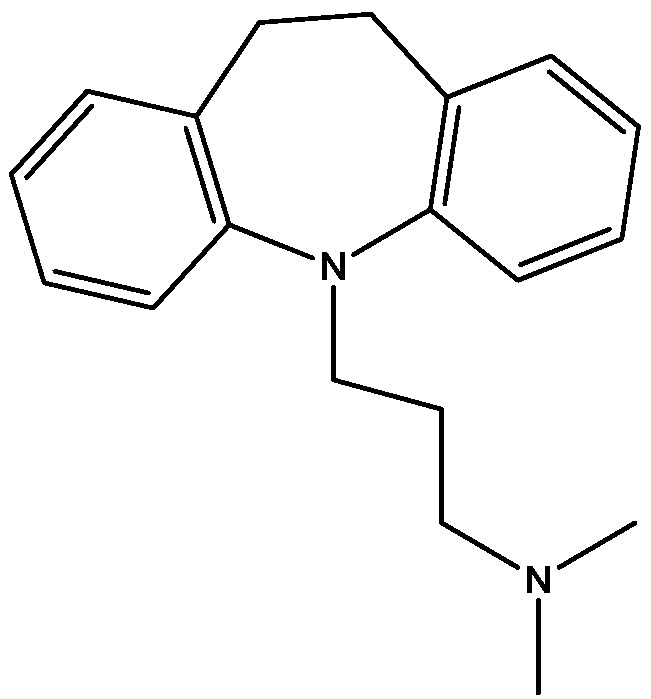
Imipramine.

Recently, many studies have suggested that the glutamatergic system, especially NMDARs, contributes to the pathophysiology of major depressive disorders [40], and they are also a target for rapid-acting antidepressants [41]. Rats were acutely or chronically treated with memantine (Figure 1) and imipramine (Figure 2) to evaluate their behavioural and molecular effects in forced-swimming test (FST, a widely used screening test to assess potential antidepressant-like effects) and open field tests by Réus *et al*. [42]. They found that the acute and chronic treatment with memantine and imipramine reduced immobility time of rats compared to the saline group, without affecting spontaneous locomotor activity (LMA). They also assessed brain-derived neurotrophic factor (BDNF) hippocampal levels in imipramine and memantine-treated rats by ELISA sandwich assay. Acute administration of memantine at higher dose (20 mg/kg), but not imipramine, increased BDNF protein levels in the rat hippocampus. Their findings further support the hypothesis that the NMDA receptor could be a new pharmacological target for the treatment of depression.

**Figure 3:**
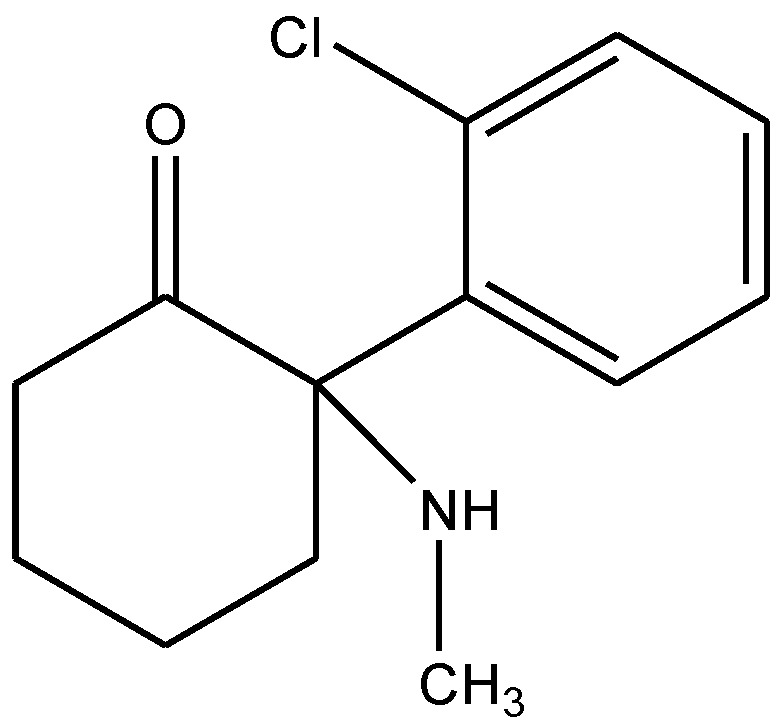
Ketamine.

Tizabi and his co-workers [6] performed the FST to determine whether ketamine (Figure 3) would exert antidepressant effects in Wistar-Kyoto (WKY) rats and whether this effect would be associated with changes in AMPA/NMDA receptor densities in the hippocampus. Acute and chronic ketamine doses were administered to adult female WKY rats and their control Wistar rats. Their LMA and immobility were evaluated in FST. They also measured hippocampal AMPA and NMDA receptor densities following chronic ketamine dose. Ketamine resulted in a dose-dependent and prolonged decrease in immobility in FST in WKY rats only, suggesting antidepressant-like effects. Chronic treatment with an effective dose of ketamine also resulted in increase in AMPA/NMDA receptor density in the hippocampus of WKY rats. LMA was not affected by ketamine treatment in either strain. Their results showed that a low ketamine dose exerts a rapid and lasting antidepressant-like effect in WKY rat model of depression. Also, they suggested that the increase in AMPA/NMDA receptor density in the hippocampus could be a contributory factor to behavioral effects of ketamine [6].

Autry and his colleagues also work on ketamine and showed that ketamine and other NMDAR antagonists produce fast-acting behavioral antidepressant-like effects in mouse models, and that these effects depend on the rapid synthesis of BDNF. They found that the ketamine-mediated blockade of NMDAR at rest deactivates eukaryotic elongation factor 2 (eEF2) kinase, resulting in reduced eEF2 phosphorylation and de-suppression of translation of BDNF. Also, they found that inhibitors of eEF2 kinase induce fast-acting behavioral antidepressant-like effects. Their findings indicate that the regulation of protein synthesis by spontaneous neurotransmission may serve as a viable therapeutic target for the discovery of fast-acting antidepressants [43].

**Figure 4:**
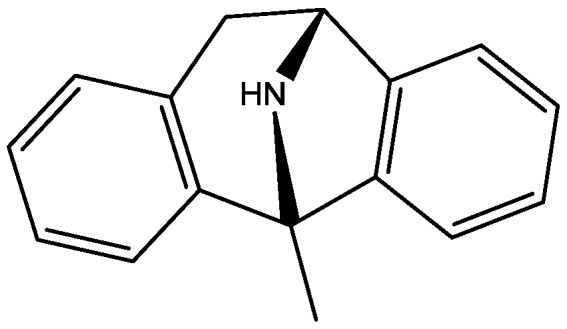
MK-801 (dizocilpine).

**Figure 5:**
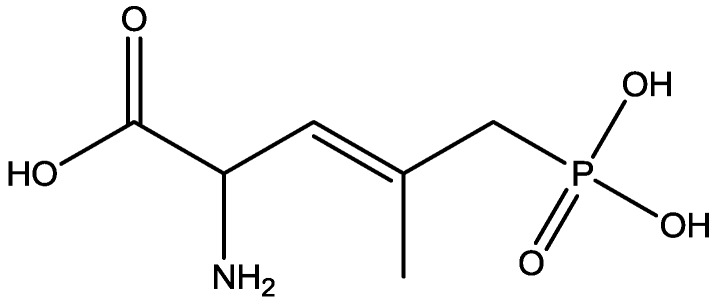
CGP 37849.

The antidepressant properties of the non-competitive NMDA receptor antagonist, MK-801 (dizocilpine, Figure 4), and the competitive antagonist, CGP 37849 (DL-(E)-2-amino-4-methyl-5-phosphono-3-pentonoic acid, Figure 5) and its (*R*)-enantiomer CGP 40116 were studied in a chronic mild stress model of depression by Papp and co-workers [40]. Animals subjected to a variety of mild stressors for a prolonged period of time showed a substantial decrease in the consumption of palatable sucrose solution (anhedonia). They found that the stress-induced deficit in sucrose intake was gradually reversed by chronic treatment with all drugs tested. They compared these results with similar administration of imipramine. The increase in sucrose intake following chronic administration of imipramine and NMDA receptor antagonists was specific to stressed animals; the behaviour of non-stressed controls was unchanged by any of the drugs tested. Their results suggest that antagonists of NMDA receptors may have antidepressant properties [44].

**Figure 6:**
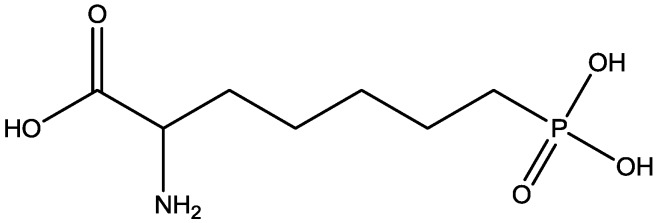
AP-7.

**Figure 7:**
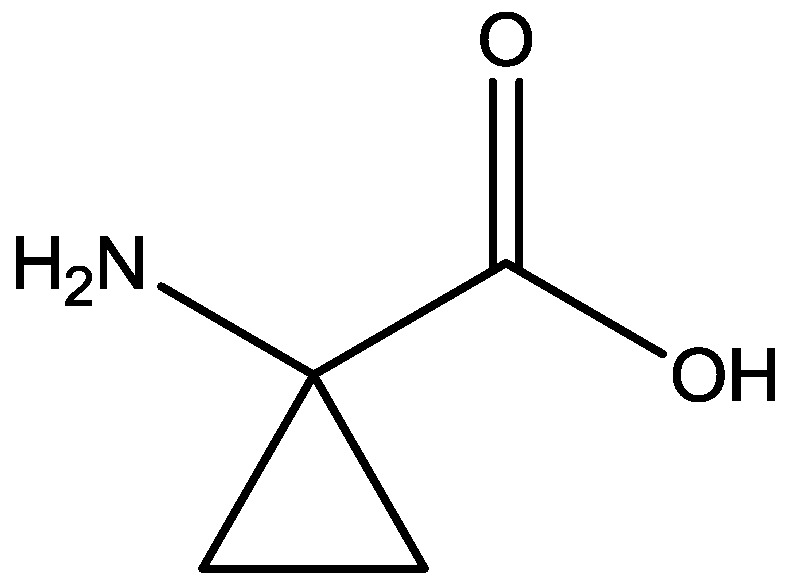
ACPC.

Inescapable stress produces a syndrome of behavioral depression sensitive to clinically effective antidepressants. Trullas and Skolnick examined the actions of functional antagonists at the NMDA receptor complex in animal models to evaluate potential antidepressants. A competitive NMDA antagonist (2-amino-7-phosphonoheptanoic acid, AP-7, Figure 6), a non-competitive NMDA antagonist (MK-801, Figure 4), and a partial agonist at strychnine-insensitive glycine receptors (1-aminocylopropanecarboxylic acid, ACPC, Figure 7) imitated the effects of clinically effective antidepressants in their models. It has been suggested that the NMDA receptor complex may be involved in the behavioral deficits induced by inescapable stress, and that substances capable of reducing neurotransmission at the NMDA receptor complex may represent a new class of antidepressants [30].

**Figure 8:**
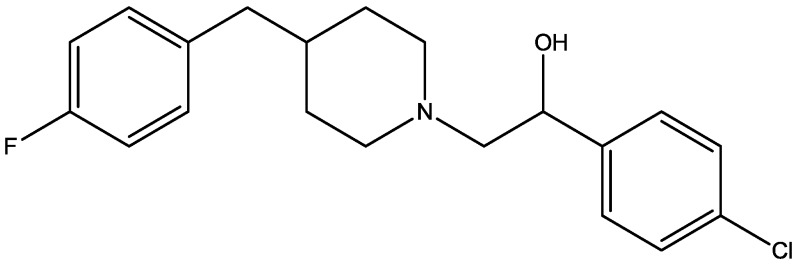
Eliprodil.

**Figure 9:**
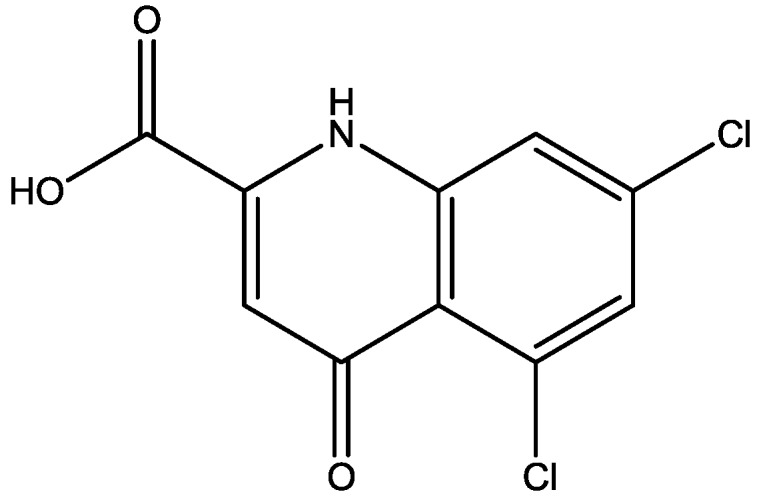
5,7-Dichlorokynurenic acid.

Eliprodil (Figure 8) is an NMDAR antagonist acting at polyamine sites. Its effects were examined in behavioral and neurochemical tests predictive of antidepressant activities by Layer *et al*. [22]. They observed that eliprodil produced a dose-dependent reduction in immobility in the FST, but was inactive in the tail suspension test in mice. Significant downregulation of β-adrenoceptors and reduction in the potency of glycine to inhibit [3*H*]-5,7-dichlorokynurenic acid (Figure 9) binding to strychnine-insensitive glycine receptors in neocortical membranes were also observed with chronic treatment of eliprodil. Their findings indicate that like other NMDAR antagonists, eliprodil possesses antidepressant-like actions in preclinical tests predictive of clinical efficacy [22].

Antidepressant-like activites of ACPC (Figure 7) and CGP 37849 (Figure 5) were examined by Przegalinski *et al*. in FST [20]. The compounds were administered intraperitoneal (i.p.) and produced dose-dependent and significant reductions of the immobility time. Similar effects were also observed after intrahippocampal (i.hp.) administration of the compunds. Imipramine (Figure 2) was used as a reference drug. It significantly shortened the immobility time after both i.p. and i.hp. administration. Their results indicate that, like imipramine, ACPC and CGP 37849 exhibit antidepressant-like activities in the FST in rats. In addition, the hippocampus may be one of the neuroanatomical sites involved in mediating these effects [20].

**Figure 10:**
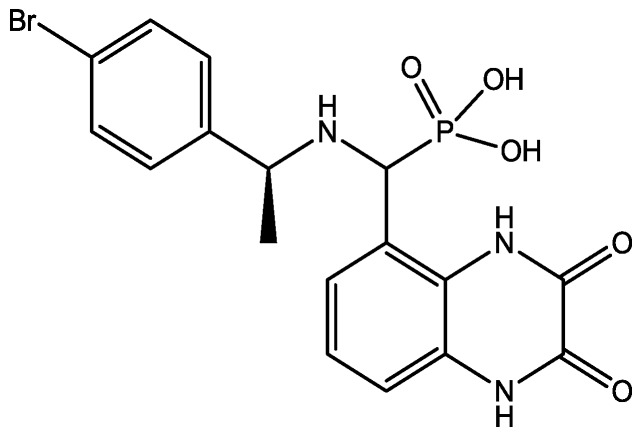
NVP-AAM077.

**Figure 11:**
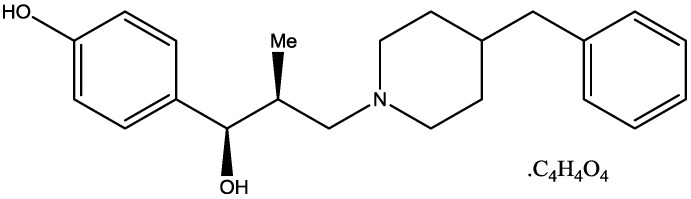
Ro 25-6981.

There are many studies that examined the role of NMDA receptors in the induction of long-term potentiation (LTP) and long-term depression (LTD) but which particular NR2 subunits are involved in these processes still remain unclear. The effects of subtype selective NMDA receptor antagonists on LTP induced by high frequency stimulation (100 Hz for 1 s) and LTD induced by low frequency stimulation (1 Hz for 15 min) were studied by Bartlett *et al.* in the CA1 region of hippocampal slices from 14 day old Wistar rats [41]. They did their experiments using NVP-AAM077 (NVP, Figure 10, 14-fold selective for NR2A over NR2B receptors) and Ro 25-6981 (Ro, Figure 11, selective for NR2B receptors). Both LTP and LTD were reduced by NVP, but only LTP was reduced by Ro. LTP was reduced by 63% at 0.1 microM NVP and was abolished at 0.4 microM whereas 5 microM Ro reduced it by 45%. These data are consistent with roles for both NR2A and NR2B in the induction of LTP under their experimental conditions. Ro (5 microM) did not affect the LTD, and NVP produced a concentration dependent inhibition of LTD which was complete at 0.4 microM. Their results showed that different NVP-sensitive NR2 subunit-containing NMDA receptors are required for LTP and LTD [45].

**Figure 12:**
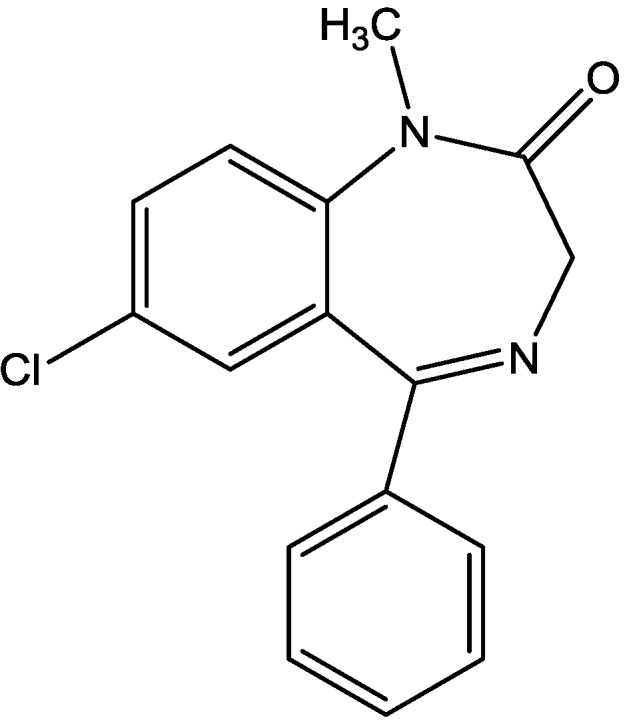
Diazepam.

**Figure 13:**
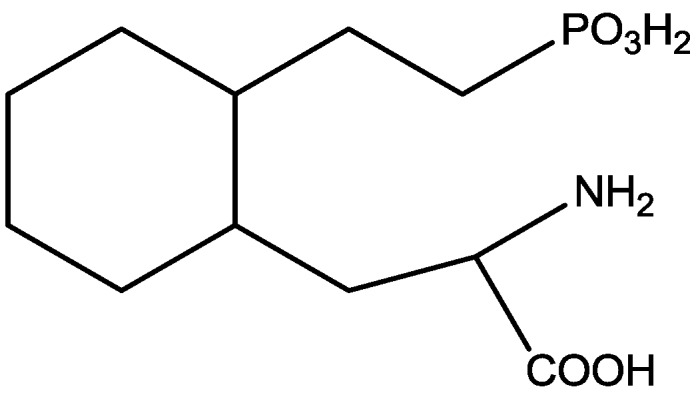
NPC 17742.

**Figure 14:**
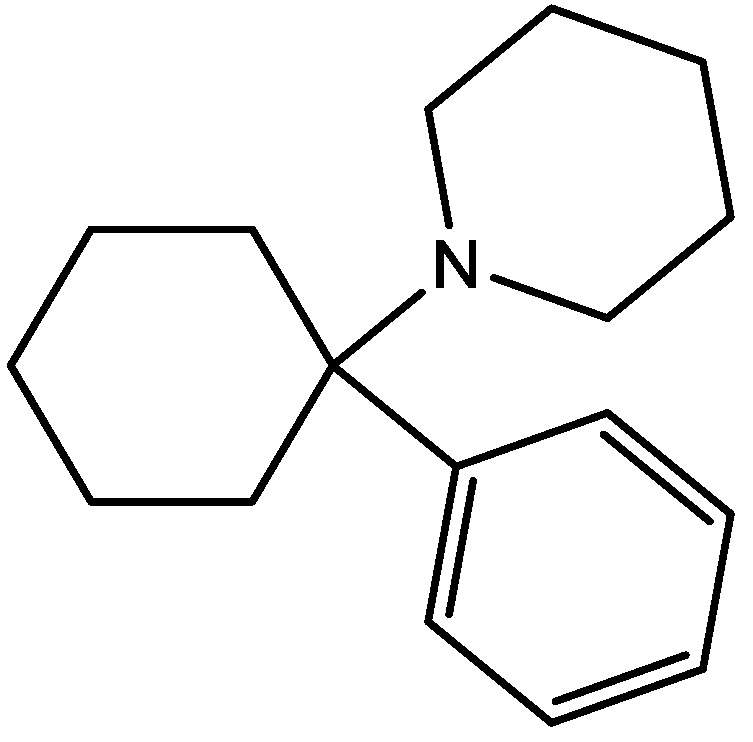
Phencyclidine.

**Figure 15:**
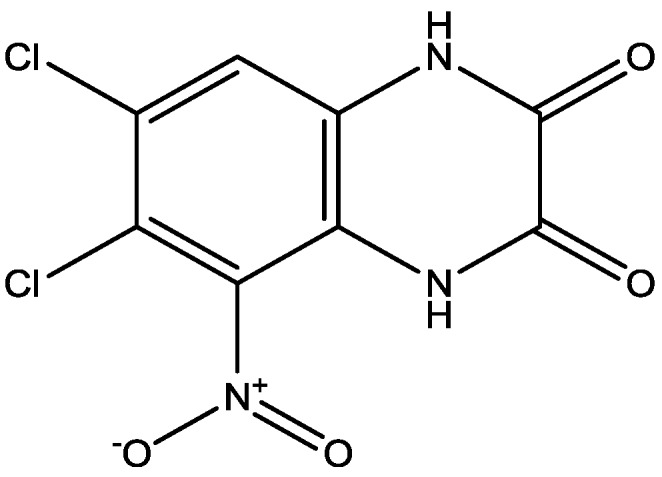
ACEA 1021.

**Figure 16:**
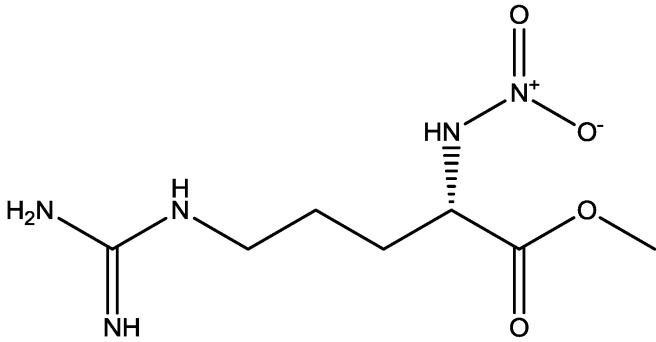
*N*-Nitro-L-arginine methyl ester.

Wiley and his colleagues examined potential anxiolytic effects of site-selective NMDA receptor antagonists. Diazepam (Figure 12), NPC 17742 [2*R*,4*R*,5*S*-2-amino-4,5-(1,2-cyclohexyl)-7-phosphono-heptanoic acid, a competitive NMDA receptor antagonist, Figure 13], phencyclidine (the phencyclidine-site NMDA receptor antagonist, Figure 14), ACEA 1021 (5-nitro-6,7-dichloro-1,4-dihydro-2,3-quinoxalinedione, a putative glycine-site antagonist, Figure 15), and *N*-nitro-L-arginine methyl ester (a nitric oxide synthase inhibitor, Figure 16) were injected to mice that were placed in the center of an elevated maze shaped like a plus sign. Two opposing arms were enclosed by high walls; the crossing arms were open. The number of entries and time spent in each type of arm were measured during 5 min tests. Results showed that diazepam and NPC 17742 increased number of open arm entries and open arm time. *N*-Nitro-l-arginine methyl ester which may interfere with the transduction of NMDA receptor activation, also increased open arm entries and time. Phencyclidine increased open arm entries but failed to increase open arm time. ACEA 1021 had significant effects only on open arm entries at the highest dose tested. Their results indicate that NMDA receptor antagonists have potential as anxiolytic agents, but that differences among antagonists acting at different sites may be expected [46].

**Figure 17:**
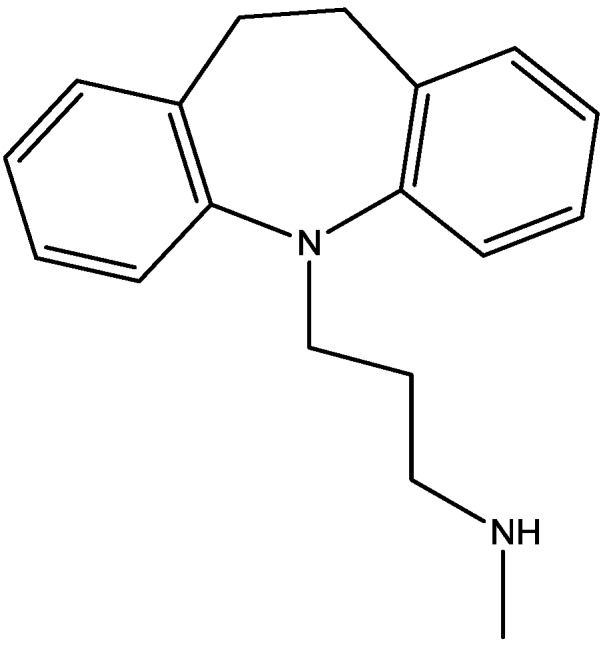
Desipramine.

**Figure 18:**
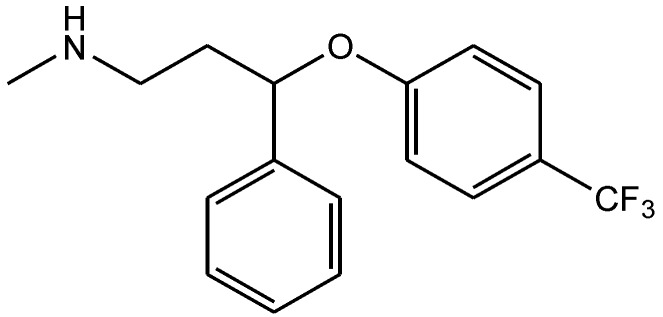
Fluoxetine.

Tricyclic desipramine (Figure 17) and the selective serotonin reuptake inhibitor fluoxetine (Figure 18) inhibit NMDARs as shown by Kiss *et al.* [25]. Since the subtypes of NMDARs are different in their physiological and pathological functions, they investigated whether the effects of antidepressants is subtype-specific. They showed that both SSRI fluoxetine and tricyclic desipramine are able to inhibit the GluN2B subunit-containing NMDA receptors in low micromolar concentration range, but fluoxetine had no effect on the GluN1/GluN2A receptor subtype. Their data suggest that the GluN2B-containing receptor subtype may be specifically involved in the pathophysiology of depression and thus the mechanism of action of antidepressants. The selective inhibitory effects of fluoxetine on GluN2B-containing receptors implies an exceptional neuroprotective potential for this drug and may be promising [25].

**Figure 19:**
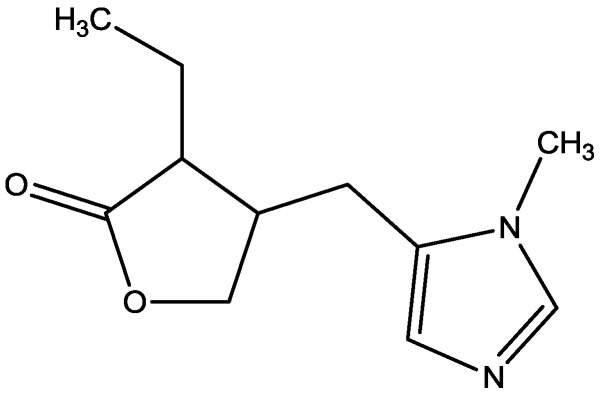
Pilocarpine.

Lopes-Aguiar and his co-workers investigated the muscarinic and glutamatergic modulation of LTD in the intact projections from CA1 to medial prefrontal cortex (mPFC) *in vivo*. They recorded the cortical-evoked responses in urethane anesthetized rats for 30 min during baseline and 4 h following LTD. One group of rats received microinjection of pilocarpine (PILO, Figure 19) immediately before or 20 min after a sub-threshold LTD protocol. Other groups received the selective NMDA receptor antagonist (AP-7, Figure 6) 10 min prior to PILO or prior to a supra-threshold LTD protocol. Their results show that PILO converts a transient cortical depression into a robust LTD, stable for at least 4 h. PILO does not change either mPFC basal neurotransmission or late LTD when applied after a sub-threshold LTD protocol. Their data also show that NMDA receptor preactivation is essential to the muscarinic enhancement of mPFC synaptic depression, because AP7 microinjection into the mPFC blocked the conversion of transient depression into long-lasting LTD produced by PILO. Also, the long-lasting LTD was effectively blocked by AP7 [47].

**Figure 20:**
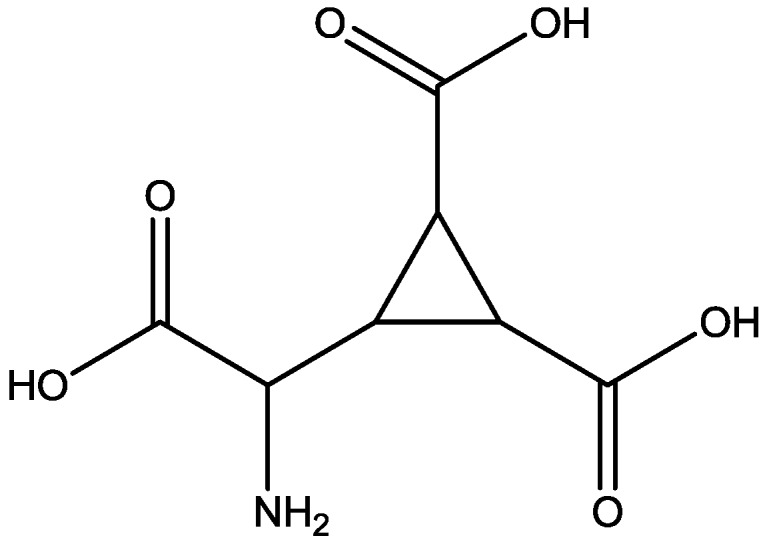
DCG-IV.

**Figure 21:**
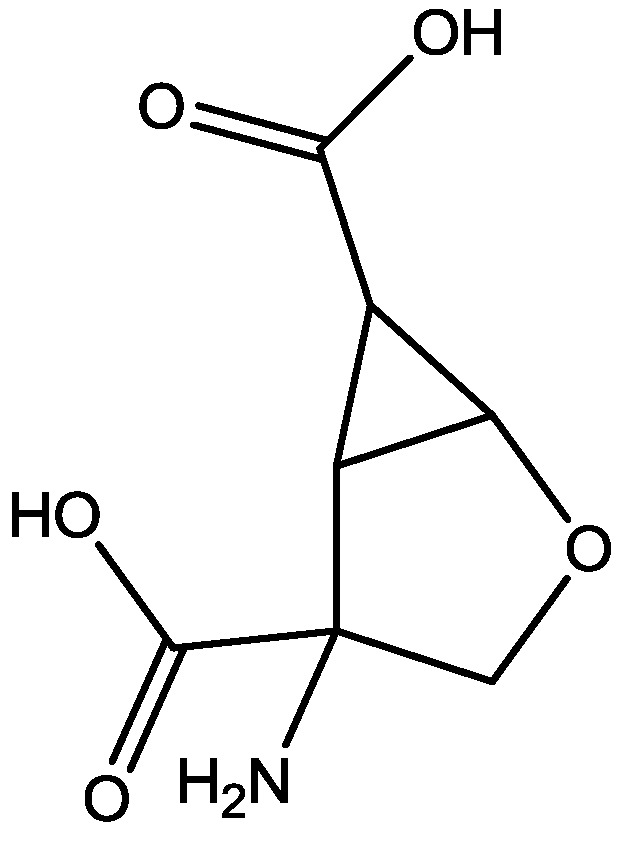
LY379268.

**Figure 22:**
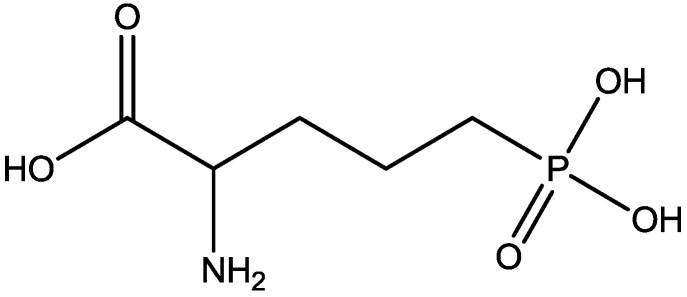
APV (D-AP5).

Studies have shown that group II metabotropic glutamate receptor (mGluR) agonist (2S,20R,30R)-2-(20,30-dicarboxycyclopropyl) glycine (DCG-IV, Figure 20) can induce LTD of excitatory transmission on layer V pyramidal neurons of rat mPFC. LTD relies on activation of both group II mGluRs and NMDARs. Huang and Hsu showed that the ability of DCG-IV to induce LTD was imitated by LY379268 (a more selective group II mGluR agonist, Figure 21). The induction of LTD by a lower concentration of DCG-IV or LY379268 was blocked by APV (a competetive NMDAR antagonist, Figure 22). High concentration of DCG-IV or LY379268 can induce LTD that is independent of synaptic NMDAR activation. Their results suggest that molecular cooperation between group II mGluRs and synaptic NMDARs may facilitate the induction of group II mGluR-mediated LTD, but enhancing group II mGluR activation may remove NMDAR involvement in this form of synaptic plasticity [48].

**Figure 23:**
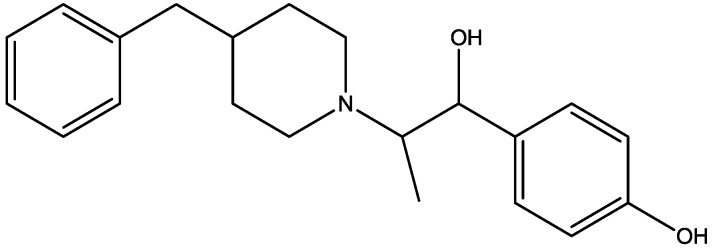
Ifenprodil.

The role of NR2B-containing synaptic NMDARs in the induction of LTD in CA1 pyramidal cells has been studied using the selective NR2B antagonists, ifenprodil (Figure 23) and Ro25-6981 (Figure 11) by Morishita *et al*. Both antagonists reduced NMDAR-mediated synaptic currents, but they did not prevent induction of LTD. These results demonstrate that activation of NR2B containing NMDARs is not an absolute requirement for the induction of LTD in the hippocampus [49].

Post-stress intrahippocampal administration of AP-7 (Figure 6) attenuated the restraint-induced decreased exploration in an elevated plus maze 24 h later. Padovan and Guimaraes [31] sought to test if this treatment would also attenuate the increased immobility seem in FST due to pre-exposure to the stressful situation. Male Wistar rats were submitted to 15 min of forced swimming and tested 24 h later. AP-7 was injected into bilateral intrahippocampal either before or after the test. Post-stress treatment increased latency to display the first episode of immobility and tended to reduce total immobility time. AP-7 was ineffective when given before stress or before test and in non-stressed animals. Their results suggest that NMDA receptors located in the dorsal hippocampus are involved in the behavioral changes observed in the FST [31].

**Figure 24:**
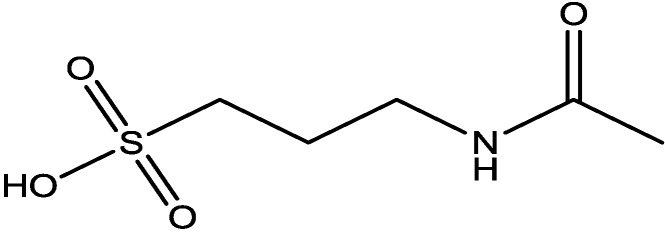
Acamprosate.

**Figure 25:**
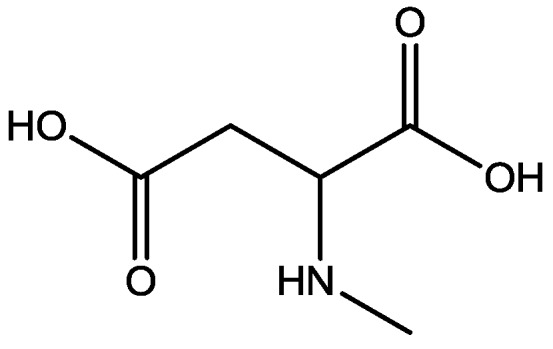
NMDA.

**Figure 26:**
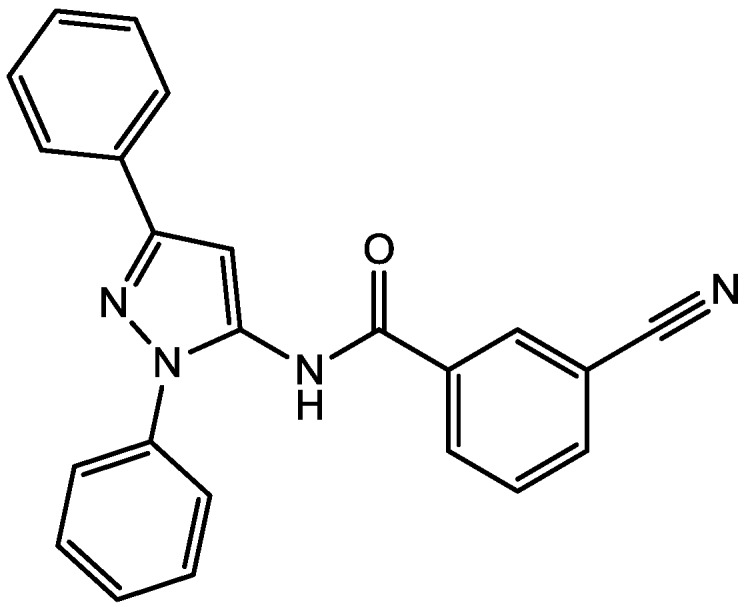
CDPPB.

Pałucha-Poniewiera and Pilc investigated potential antidepressant-like effects of acamprosate (3-acetamidopropane-1-sulfonic acid, Figure 24), NMDA (Figure 25) and mGlu5 receptor antagonist, CDPPB (Figure 26). They found potential antidepressant-like effects of acamprosate at doses of 100–400 mg/kg in the tail suspension test (TST) in mice. Moreover, they showed that the antidepressant-like effects of acamprosate used at a dose of 200 mg/kg was dependent on NMDA and mGlu5 receptor blockade, because NMDA and mGlu5 receptor positive allosteric modulator, CDPPB [3-cyano-*N*-(1,3-diphenyl-1*H*-pyrazol-5-yl) benzamide, 3 mg/kg], antagonized its activity in the TST. Their data suggest that acamprosate may induce antidepressant-like effects and NMDA and mGlu5 receptors may be crucial targets of acamprosate in this action [50].

**Figure 27:**
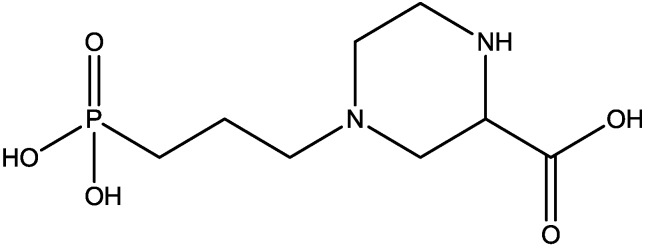
CPP.

Changes in curvature of the post synaptic density (PSD) and apposition zone (AZ) are important to determining synaptic efficacy. Medvedev and co-workers [51] have examined curvature of PSDs and AZs 24 h following homosynaptic LTP, and heterosynaptic LTD *in vivo*, in awake adult rats. Curvature changes were analyzed using three dimensional (3-D) reconstructions of electron microscope images of ultrathin serial sections. Very large and significant changes in 3-D measurements of AZ and PSD curvature occurred 24 h following both LTP and LTD. The changes in curvature of mushroom and thin spine PSDs and apposition zones were blocked by 3-[(R)-2-Carboxypiperazin-4-yl]-propyl-1-phosphonic acid (CPP, Figure 27, an NMDA receptor antagonist). They examined the effects of CPP alone on PSD and apposition zone curvature to determine whether these changes resulted from effects of the NMDAR antagonist or from its coincidence with synaptic activation during testing. They observed that CPP alone also caused a small decrease in curvature of both PSD and apposition zone of mushroom and thin spines [51].

Hrabetova and Sacktor also examined the effects of CPP and D-2-amino-5-phosphonovaleric acid (D-AP5, Figure 22, a competitive antagonist) on the induction of LTP and LTD. CPP binds with high affinity to conventional NMDA receptors subtypes, but not to atypical subtypes. LTP, LTD, and depotentiation were all blocked by applications of D-AP5, and only LTP was blocked by CPP. Their observations suggest that decreases and increases of synaptic strength are mediated by the activation of distinct NMDA receptor subpopulations [52].

**Figure 28:**
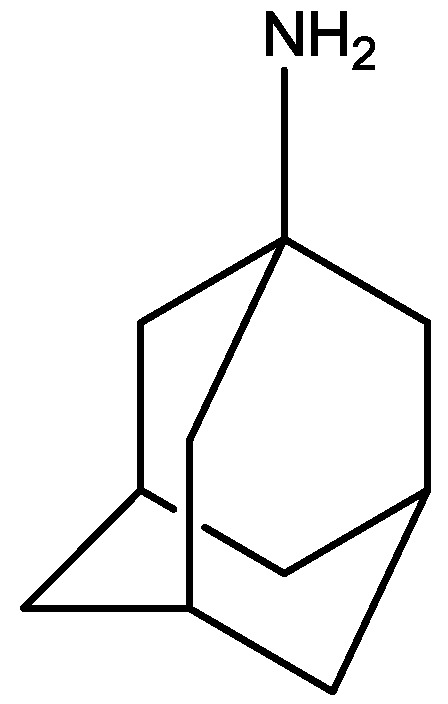
Amantadine.

**Figure 29:**
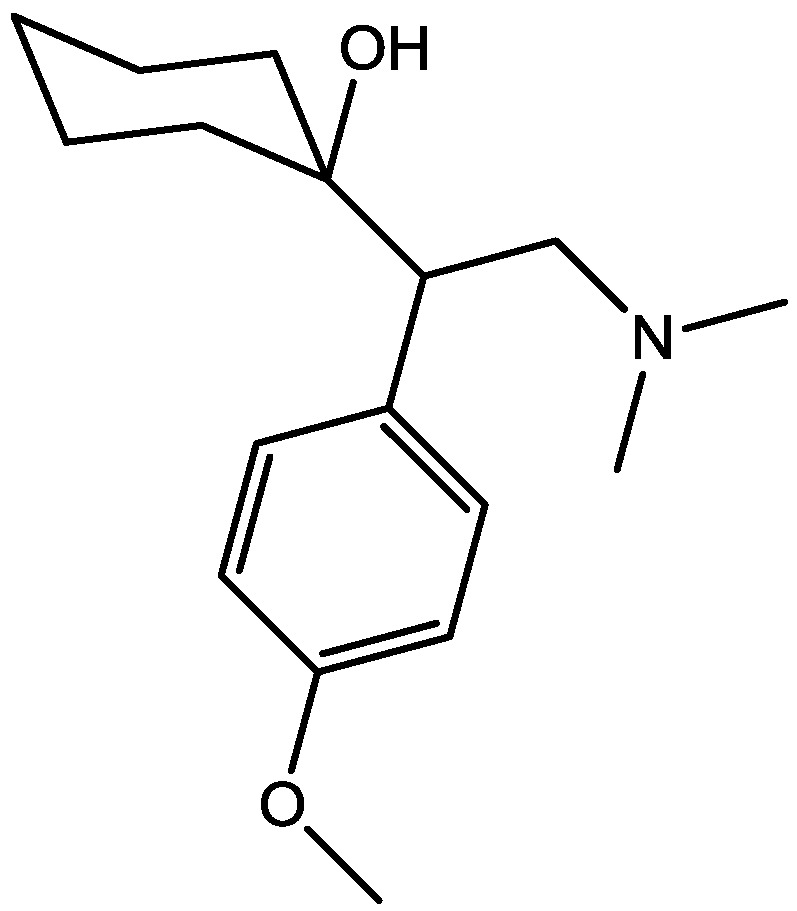
Venlafaxine.

**Figure 30:**
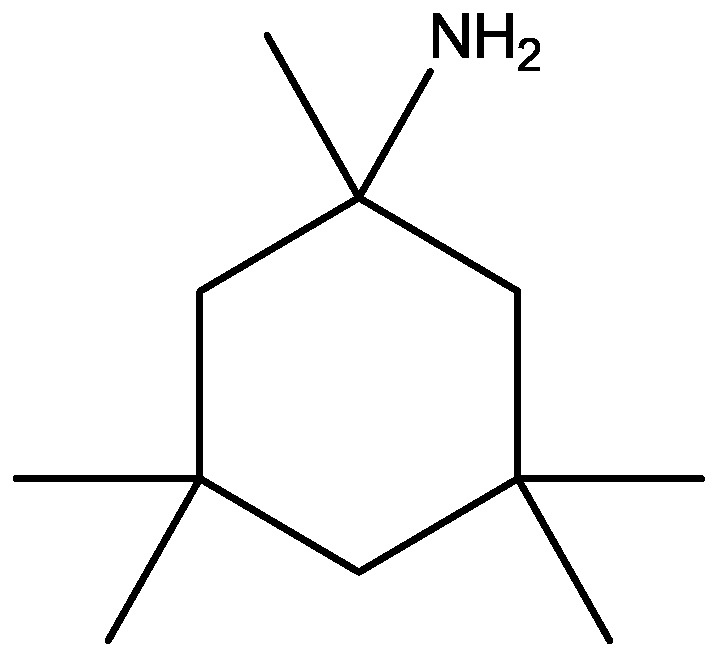
Neramexane.

Rogoz and co-workers have reported that administration of imipramine (Figure 2) and amantadine (an uncompetitive NMDA receptor antagonist, Figure 28) reduced immobility time in the FST in rats to a much greater extent than either treatment alone. They also investigated the possibility of synergistic interactions between three antidepressants [imipramine, venlafaxine (Figure 29), fluoxetine (Figure 18)] with three uncompetitive NMDA receptor antagonists [amantadine, memantine (Figure 1) and neramexane (Figure 30)]. Most combinations resulted in synergistic antidepressive-like effects in the FST. Fluoxetine was inactive when given alone, showed a positive effect when combined with amantadine, memantine or neramexane. Their results suggest that the combination of traditional antidepressant drugs and NMDA receptor antagonists may produce enhanced antidepressive effects, and this is of particular relevance for antidepressant-resistant patients [53].

**Figure 31:**
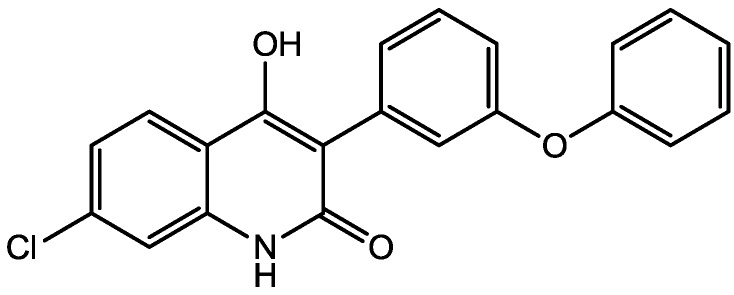
L-701,324.

**Figure 32:**
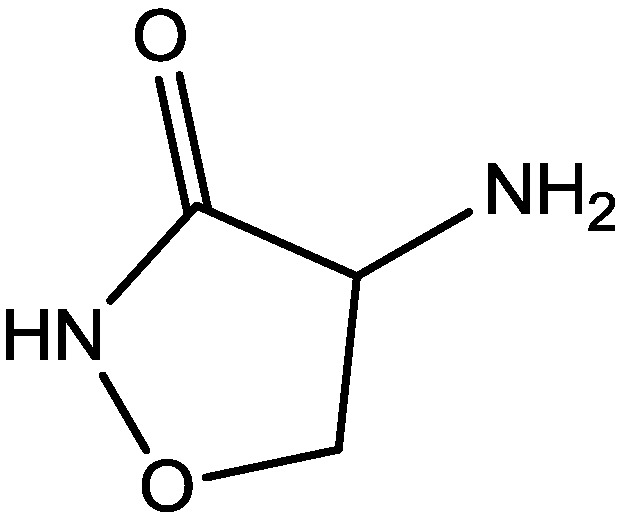
D-Cycloserine.

It has been demonstrated that magnesium shows antidepressant-like activity in FST. Also, it has alo been shown that administration of magnesium with antidepressants enhance this activity. Mechanism involved in such activity is still unclear. Poleszak and co-workers have examined the involvement of NMDA/glutamate pathway in the magnesium activity in FST in mice. They investigated the influence of NMDA antagonists with sub-effective doses of magnesium. They observed that magnesium-induced antidepressant-like activity was antagonized by NMDA. When low, ineffective doses of CGP 37849 (Figure 5), L-701,324 (Figure 31), d-cycloserine (Figure 32), and MK-801 (Figure 4) were administered with low and ineffective doses of magnesium, significant reduction of immobility time in FST was observed. Their study indicates the involvement of NMDA/glutamate pathway in the antidepressant-like activity of magnesium in mouse and further suggests antidepressant properties of magnesium [54].

**Figure 33:**
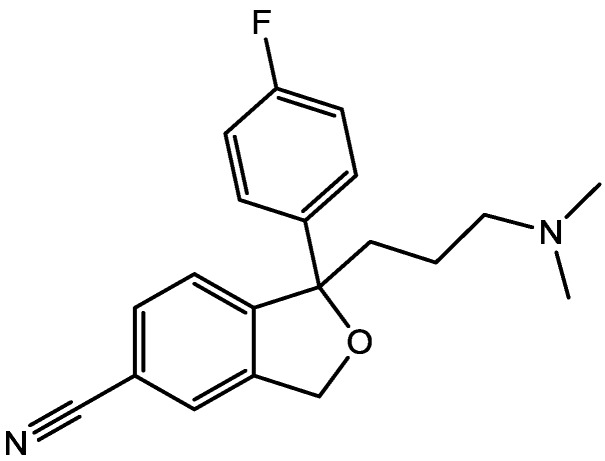
Escitalopram.

**Figure 34:**
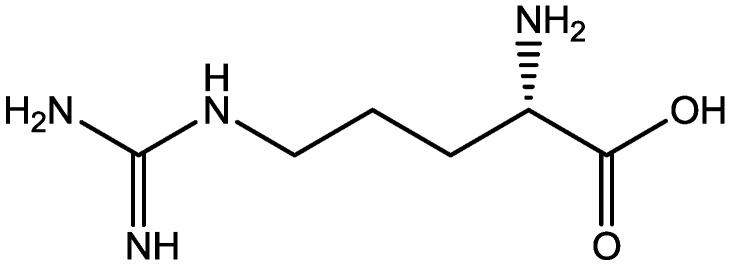
L-Arginine.

**Figure 35:**
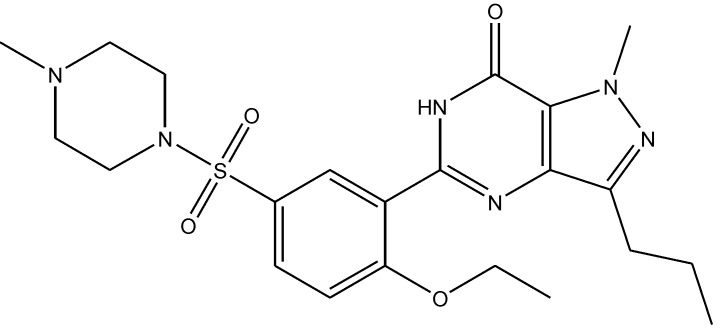
Sildenafil.

**Figure 36:**
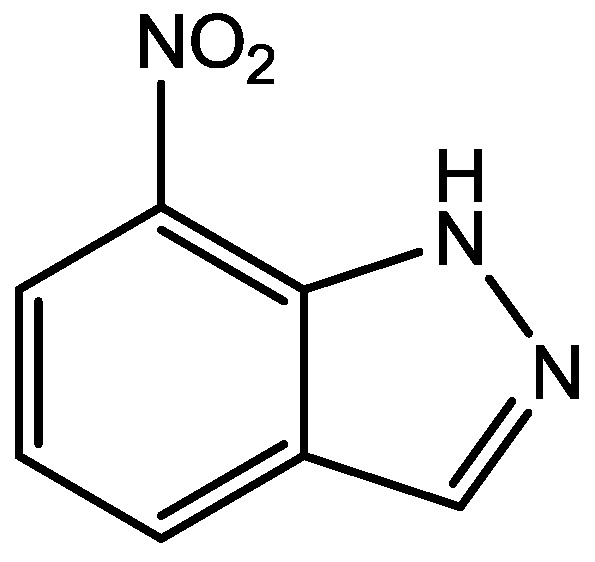
7-Nitroindazole.

**Figure 37:**
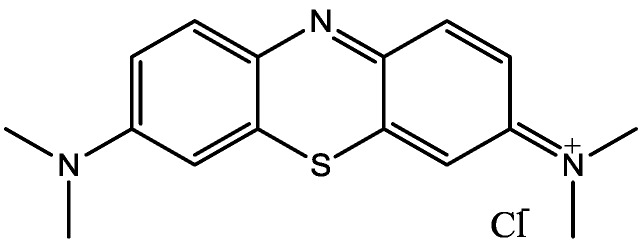
Methylene blue.

**Figure 38:**
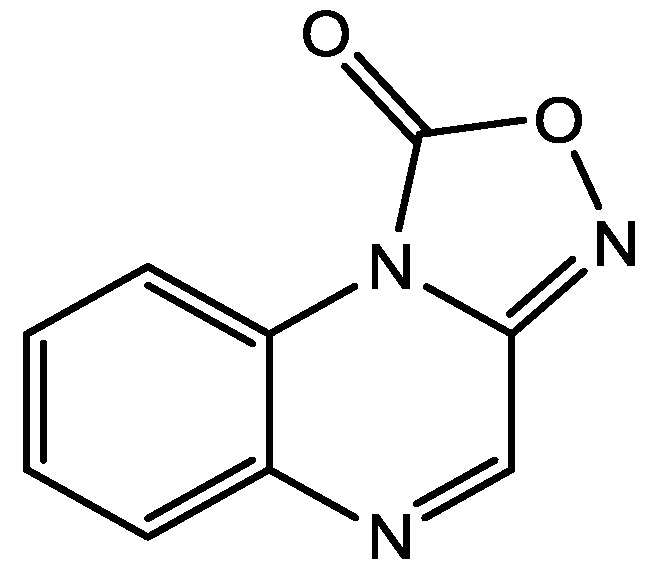
ODQ.

Zomkowski and his colleques investigated the effect of escitalopram (Figure 33), a serotonin reuptake inhibitor used in the treatment of depression and anxiety, in FST in mice. They tested the hypothesis that the inhibition of NMDA receptors and NO-cGMP synthesis are implicated in its mechanism of action in FST. Administration of escitalopram by i.p. and p.o. routes reduced the immobility time in FST. Pretreatment of mice with NMDA, L-arginine (a substrate for nitric oxide synthase, Figure 34) or sildenafil (phosphodiesterase 5 inhibitor, Figure 35) prevented the antidepressant-like effects of escitalopram in the FST. Combination of escitalopram with the administration of 7-nitroindazole (a neuronal nitric oxide synthase inhibitor, Figure 36), methylene blue (an inhibitor of both nitric oxide synthase and soluble guanylate cyclase, Figure 37) or ODQ (a soluble guanylate cyclase inhibitor, Figure 38) reduced the immobility time in the FST as compared with either drug alone. Their data suggest that the antidepressant-like effects of escitalopram is dependent on inhibition of either NMDA receptors or NO-cGMP synthesis [55].

**Figure 39:**
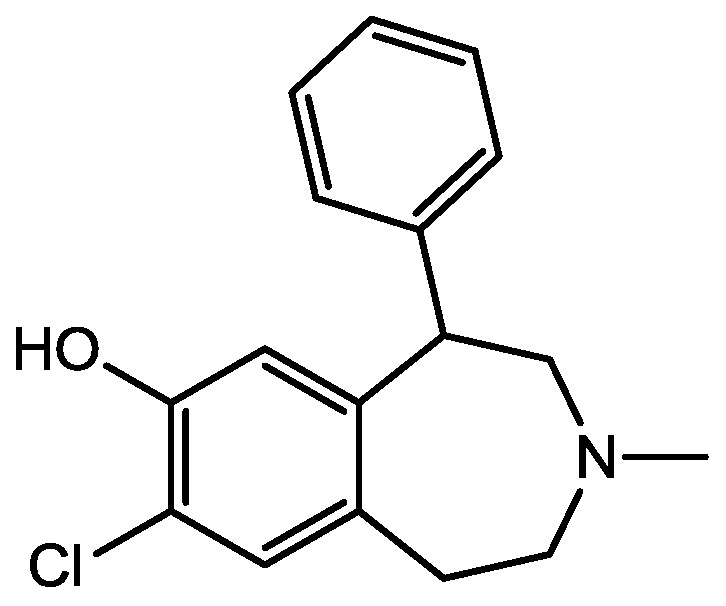
SCH 23390.

**Figure 40:**
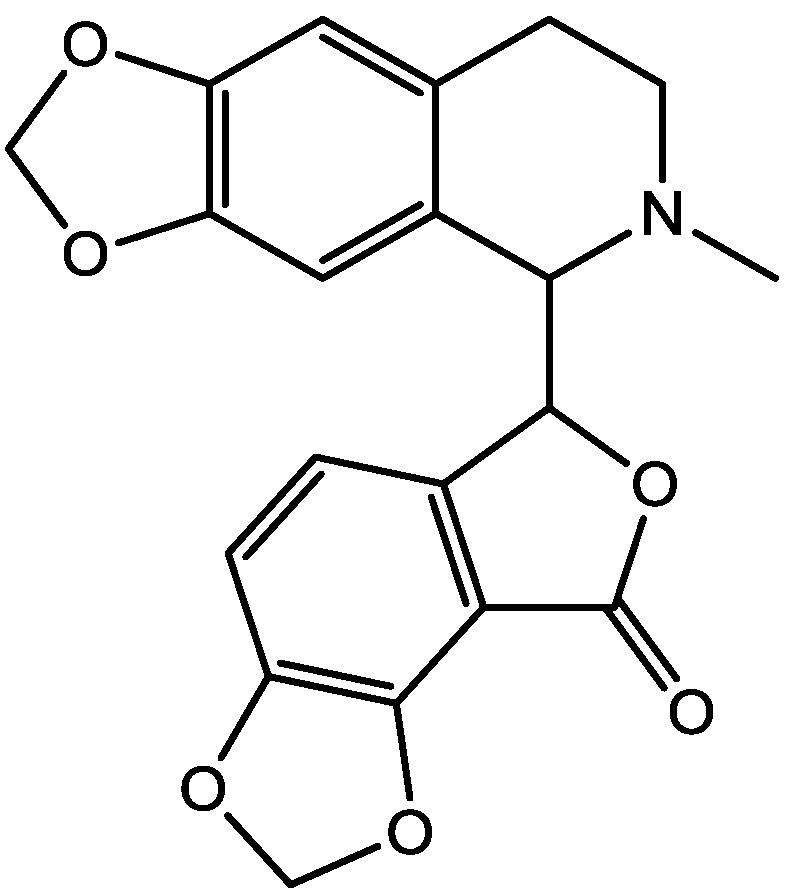
Bicuculline.

**Figure 41:**
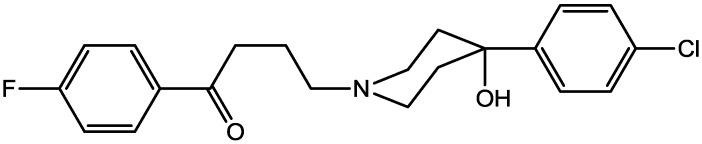
Haloperidol.

Ketamine (Figure 3) has psychotomimetic actions but the detailed mechanisms have not been established. To elucidate neural mechanisms of the effects of ketamine (at doses that exert psychotomimetic effects without anesthetic and analgesic effects), cortical synaptic responses were evaluated *in vivo* by Kamiyama *et al*. [56]. Ketamine dose-dependently decreased hippocampal stimulation-evoked potential in the mPFC in rats. The psychotomimetic effects observed in ketamine-treated groups are associated with the induction of synaptic depression in the hippocampus-mPFC neural pathway. They also examined the underlying mechanisms of the ketamine induced synaptic depression under anesthesia. They observed that ketamine-induced synaptic depression was blocked with pretreatment of SCH 23390 (a dopamine D1 receptor antagonist, Figure 39) or bicuculline (a GABA_A_receptor antagonist, Figure 40), but not with haloperidol (a dopamine D2 receptor antagonist, Figure 41). Their findings suggest that hippocampus-mPFC synaptic transmission is depressed by ketamine at the dose that exerts psychotomimetic symptoms through mechanisms involving dopaminergic modulation mediated via D1 receptors, which may be augmented by synaptic inhibition mediated via GABA_A_ receptors [56].

Preskorn and his co-workers conducted a randomized, placebo-controlled, double-blind study to evaluate the antidepressant efficacy, safety, and tolerability of a GluN2B (NR2B) subunit-selective NMDAR antagonist, CP-101,606. Subjects had major depression, and study had two treatment periods. a 6-week open-label trial of paroxetine and a single-blind, intravenous placebo infusion were administered (period 1), and then a randomized double-blind single infusion of CP-101,606 or placebo plus continued treatment with paroxetine for up to an additional 4 weeks were received by period 1 non-responders (period 2). Montgomery-Åsberg Depression Rating Scale and 17-item Hamilton Depression Rating Scale was used to assess depression severity. Hamilton Depression Rating Scale response rate was 60% for CP-101,606 *versus* 20% for placebo. More than 70% of CP-101,606-treated subjects continued response status for at least 1 week after the infusion. They stated that CP-101,606 was safe, generally well tolerated, and capable of producing an antidepressant response without also producing a dissociative reaction [57].

**Figure 42:**
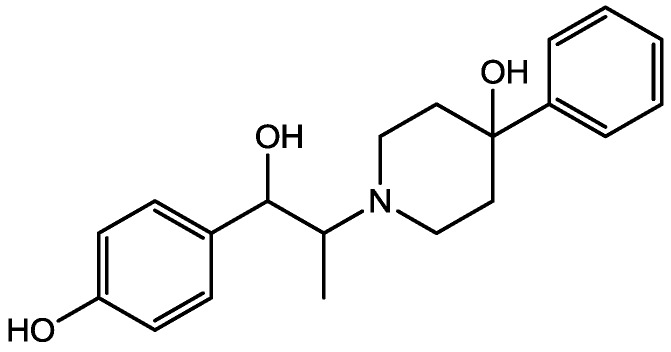
CP-101, 606.

The observations described in this and similar works are leading to new interests by us and others in the possibilities of discovery of NMDAR antagonists with reduced toxicities as potential compounds for treatment of depression and other CNS disorders [58].

## 4. Conclusions

The *N*-methyl-D-aspartate receptor (NMDAR) subtype of glutamate receptors has been implicated in crucial pathophysiological processes such as schizophrenia, major depression, and post-traumatic stress disorder [58]. In this review, we summarized studies from various laboratories demonstrating that NMDA receptor antagonists exert antidepressant like effects and augment such properties for known antidepressant compounds in preclinical animal models. The recent findings showing ketamine to be effective clinically in major depression is very encouraging. The main challenge is discovery of compounds with more tolerable side effect profiles. Thus, future studies could lead to novel compounds involving NMDAR mechanisms and which could be useful in the treatment of a variety of neuropsychiatric disorders.
